# The endothelial dysfunction blocker CU06-1004 ameliorates choline-deficient L-amino acid diet-induced non-alcoholic steatohepatitis in mice

**DOI:** 10.1371/journal.pone.0243497

**Published:** 2020-12-04

**Authors:** Cho-Rong Bae, Haiying Zhang, Young-Guen Kwon

**Affiliations:** 1 Department of Biochemistry, College of Life Science and Biotechnology, Yonsei University, Seoul, Republic of Korea; 2 CURACLE Co., Ltd., Seongnam-si, Gyeonggi-do, Republic of Korea; Faculty of Medicine & Health Science, UNITED ARAB EMIRATES

## Abstract

Non-alcoholic steatohepatitis (NASH) is a severe, advanced form of non-alcoholic fatty liver disease (NAFLD) that is associated with features of metabolic syndrome and characterized by hepatic steatosis, inflammation, and fibrosis. In addition, NASH is associated with endothelial dysfunction within the hepatic vasculature. Treatment with CU06-1004 (previously called Sac-1004) ameliorates endothelial dysfunction by inhibiting hyperpermeability and inflammation. In this study, we investigated the protective effects of CU06-1004 in a choline-deficient L-amino acid (CDAA)-induced mouse model of NASH for 3 or 6 weeks. Specifically, we evaluated the effects of CU06-1004 on lipid accumulation, inflammation, hepatic fibrosis, and liver sinusoidal endothelial cell (LSEC) capillarization through biochemical analysis, immunohistochemistry, and real-time PCR. We found that the administration of CU06-1004 to mice improved liver triglyceride (TG) and serum alanine aminotransferase (ALT) in this CDAA-induced model of NASH for 6 weeks. In groups of NASH induced mice for both 3 and 6 weeks, CU06-1004 significantly reduced the hepatic expression of genes related to lipogenesis, inflammation, and cell adhesion. However, expression of genes related to hepatic fibrosis and vascular endothelial changes were only decreased in animals with mild NASH. These results suggest that the administration of CU06-1004 suppresses hepatic steatosis, inflammation, fibrosis, and LSEC capillarization in a CDAA-induced mouse model of NASH. This suggests that CU06-1004 has therapeutic potential for the treatment of mild NASH.

## Introduction

Non-alcoholic fatty liver disease (NAFLD) is a broad term covering a range of chronic liver diseases and is a major cause of conditions related to metabolic syndrome, including obesity, type 2 diabetes, and insulin resistance [1]. Non-alcoholic steatohepatitis (NASH) is an advanced form of NAFLD and typically presents with steatosis, inflammation, and fibrosis in the liver [2]. However, although several studies have tested various treatment approaches for NASH, there is no approved, effective therapy available to date [3–5].

The vascular endothelium is the key barrier between the systemic circulation and tissues [6]. The liver endothelium is primarily composed of liver sinusoidal endothelial cells (LSECs) [7], which help maintain liver homeostasis and contribute to efficient nutrient and gas exchange [8]. Liver injury often begins with damage to the LSECs, and the importance of LSEC dysfunction in the pathogenesis of NAFLD and NASH has been described in detail [9, 10]. After liver injury, LSECs increased sinusoidal capillarization and acquire pro-inflammatory and pro-fibrotic features [11–13]. Therefore, preventing LSEC capillarization may be an important factor in the treatment of NASH.

Treatment with CU06-1004, a previously known as Sac-1004, ameliorates endothelial dysfunction and enhances endothelial barrier function through the cAMP/Rac/cortactin pathway in human vesicular endothelial cells (HUVECs) [14]. It has anti-hyperpermeability and anti-inflammatory functions that help protect against EC dysfunction-related diseases such as cerebral ischemia, cancer, and diabetes [15–17]. However, the effects of CU06-1004 in animal models of NASH remain unclear. The choline-deficient, L-amino acid-defined (CDAA) diet-induced mouse model of NASH is commonly used to study aggressive NASH. Mice on the CDAA diet reliably develop hepatic steatosis, inflammation, liver fibrosis, and insulin resistance [18]. Indeed, mice fed a CDAA diet for 3 weeks typically have increased liver weight, hepatic triglyceride, inflammation, and mild fibrosis [19]. In addition, CDAA diet induced mice for 6 weeks were developed increased fatty liver with fibrosis score than CDAA diet induced mice for 3 weeks [18, 20]. In this study, we investigated the effects of oral treatment with CU06-1004 on hepatic steatosis, inflammation, fibrosis, and LSEC capillarization using a CDAA-induced mouse model of NASH for 3- or 6-weeks.

## Materials and methods

### Animals and experimental design

Seven-week-old male mice were purchased from DBL (Daehan Biolink, Seoul, Korea). The animals were maintained on a control diet (Purina Laboratory rodent diet 38057; Purina Korea Inc., Seoul, Korea) for 1 week and then separated into four groups (n = 10 per groups): control diet (Con), CDAA diet (CDAA; A06071302; Research Diets Inc., New Brunswick, NJ), and CDAA with CU06-1004 at 10 mg/kg/day for 3 or 6 weeks. All mice were housed in standard cages at constant temperate (23°C) and humidity (60%) with a 12:12-h light:dark cycle and unrestricted access to feed and water. Mice were monitored daily and weighed once per weeks. Mice were fasted for 16 h before being euthanized for tissue collection. After 3 or 6 weeks all groups were sacrificed by CO_2_ asphyxiation. No unexpected deaths of mice were observed for experiment period. All experiments involving animals were approved in advance by the Animal Care and Use Committee of the Yonsei University (Seoul, Korea) and were performed in accordance with approved guidelines (IACUC-A-201901-854-02).

### Drug treatment

CU06-1004 was synthesized as described previously [14]. Briefly, CU06-1004 was synthesized via tetrahydropyran deprotection and subsequent glycosidation with 4,6-di-O-ace-tyl-2,3-didieoxyhex-2-enopyran, in the presence of an acid. A stock solution of CU06-1004 (50 mg/mL) was prepared in dimethyl sulfoxide (DMSO) and dilutions were made in phosphate buffered saline (PBS). Mice in the 3- and 6-week groups respectively received a CDAA diet for 1 and 3 weeks, followed by 2 and 3 weeks of treatment with CU06-1004 along with the CDAA diet. Mice were treated with an equal volume of oral control solution, vehicle, or CU06-1004 (10 mg/kg body weight) once daily.

### Glucose tolerance test

One week before the end of the experiment, an intraperitoneal glucose tolerance test (GTT, oral glucose at 1 g/kg body weight) was performed in mice after a 16-h fast. Blood samples were collected 0, 15, 30, 60, and 120 min after glucose injection and measured with a glucometer (Roche, Germany, Accu chek).

### Serum and liver biochemical analyses

Triglycerides and total cholesterol were extracted from the liver tissue with chloroform-methanol (2:1, vol:vol), as previously described [21]. Briefly, chloroform-methanol was added to homogenized liver tissue, vortexed, and centrifuged; the lower phase was collected and evaporated at room temperature under a fume hood. The resulting semi-dried pellets were then dissolved in 1% Triton X-100 (VWR, USA). The hepatic and serum triglycerides (TG) and total cholesterol (TC) contents were quantified using commercial kits (Asan Pharmaceutical Co., Seoul, Korea, #AM157S-K, #AM202-K). Hepatic functional parameters such as alanine aminotransferase (ALT) and aspartate transaminase (AST) activities were measured using commercial kits (Asan Pharmaceutical Co., Seoul, Korea, #AM102-K, #AM103-K). Serum concentrations of TNF-α and IL-6 were determined using Quantikine ELISA kits (R&D Systems, Minneapolis, MN, USA, #MTA00B, #M6000B).

### Histology and immunohistochemical analysis

Four to eight mice from each group were randomly selected. Livers were fixed in 4% paraformaldehyde in PBS (Sigma, Steinheim, Germany) at room temperature for 48 h, embedded in paraffin, sectioned at 6 μm, and stained with hematoxylin and eosin (H&E). Liver fibrosis was assessed by staining with a Picro Sirius Red kit (Abcam, Cambridge, MA, USA, #ab150681). For immunohistochemistry, paraffin-embedded sections were stained with antibodies against F4/80 (1:60; AbD Serotec, Oxford, UK, #MCA497GA), α-smooth muscle actin (α-SMA, 1:300, Abcam, #ab7817), ICAM-1 (1:200; Santa Cruz, CA, USA, #sc8439), and CD31 (1:200, R&D System, MN, USA, #AF3628). Images were using an eclipse microscope (Nikon, Tokyo, Japan), and the Sirius Red, F4/80, α-SMA, ICAM-1, and CD31-positive areas were quantified using Image J.

### RNA isolation and quantitative RT-PCR analysis

Out of 10 mice from each group, one mice used for representative photographs images of liver in each group, and then 9 mice used to real-time PCR. Total RNA was isolated from the liver with easy-BLUE (iNtRON, Seongnam, Korea) and cDNA was synthesized using Moloney murine leukemia virus (M-MLV) reverse transcriptase (Promega, Madison, WI, USA). Quantitative real-time polymerase chain reaction (qRT-PCR) was performed using gene-specific primers (Table 1) and SYBR Green (Invitrogen) in a Bio-Rad RT-PCR detection system. Gene expression was calculated using the 2^-ΔΔCt^ method [22]. The level of ribosomal protein 36B4 mRNA was used for normalization.

**Table 1:** Sequence of primers used for real-time quantitative PCR.

Gene	Forward	Reverse
ACC	CCCATCCAAACAGAGGGAAC	CTGACAAGGTGGCGTGAAG
CD31	CCAAAGCCAGTAGCATCATGGTC	GGATGGTGAAGTTGGCTACAGG
Col1α	AGTAACGTCGTCGTGCCTAGCAACAT	GAATACTGAGCAGCAAAGTTCCCAG
Col4α	CCAGGATGCAACGGTACAAA	ACGTGGCCGAGAATTTCAC
E-selectin	AGATACTTTCGGAAGAAAGCAAAGAA	GTAAGAAGGCACATGGTAGTTTTCAA
FAS	GCTGCTGTTGGAAGTCAGC	AGTGTTCGTTCCTCGGAGTG
ICAM-1	CGTGTGCCATGCCTTTAGCT	TCCAGTTATTTTGAGAGTGGTACAGTACTG
IL-1β	AGCACCTTCTTTCCCTTCATCTTT	GAGGTGGAGAGCTTTCAGTTCATA
MCP-1	GGCTCAGCCAGATGCAGTTAA	AGCCTACTCATTGGGATCATCTT
PPARγ	AGGCCGAGAAGGAGAAGCTGTTG	TGGCCACCTCTTTGCTCTGCTC
SREBP-1c	TTCCTCAGACTGTAGGCAAATCT	AGCCTCAGTTTACCCACTCCT
TGF-β	CAACTACTGCTTCAGCTCCACAGAG	CAAGGACCTTGCTGTACTGTGTGTC
TNF-α	TGGCCCAGACCCTCACACTCAGATC	GCCTTGTCCCTTGAAGAGAACCTGG
VCAM-1	CCCTGAATACAAAACGATCGC	CAGCCCGTAGTGCTGCAAG
36B4	TCATTGTGGGAGCAGACAATGTGG	AGGTCCTCCTTGGTGAACACAAAG

ACC, acetyl CoA carboxylase; CD31, cluster of differentiation; Col1α, collagen 1α; Col4α, collagen 4α; FAS, fatty acid synthase; ICAM-1, intercellular adhesion molecule 1; IL-1β, interleukin-1β; MCP1, monocyte chemoattractant protein-1; PPARγ, peroxisome proliferator-activated receptor γ; SREBP-1c, sterol regulatory element binding protein-1c; TGF-β, transforming growth factor-β; TNF-α, tumor necrosis factor-α; VCAM-1, vascular cell adhesion protein-1.

### Hydroxyproline assay

Five mice from each group were randomly selected. To assess liver collagen contents, hydroxyproline levels were measured with a colorimetric assay kit (Bio Vision, Milpitas, CA). Absorbance was measured at 560 nm with a microplate reader (BMG Labtech, Ortenberg, Germany, #K555-100).

### Statistical analysis

All values were expressed as mean ± SEM. Data were analyzed using SPSS software (version 12.0 for Window, IBM, Armonk, NY, USA). Statistical significance was evaluated using Student's *t*-test and one-way ANOVA with *post hoc* Tukey-Kramer test. Differences were considered significant when *P* values were *< 0*.*05*.

## Results

### Effects of CU06-1004 on metabolic parameters in CDAA-induced NASH mice

**Fig 1:**
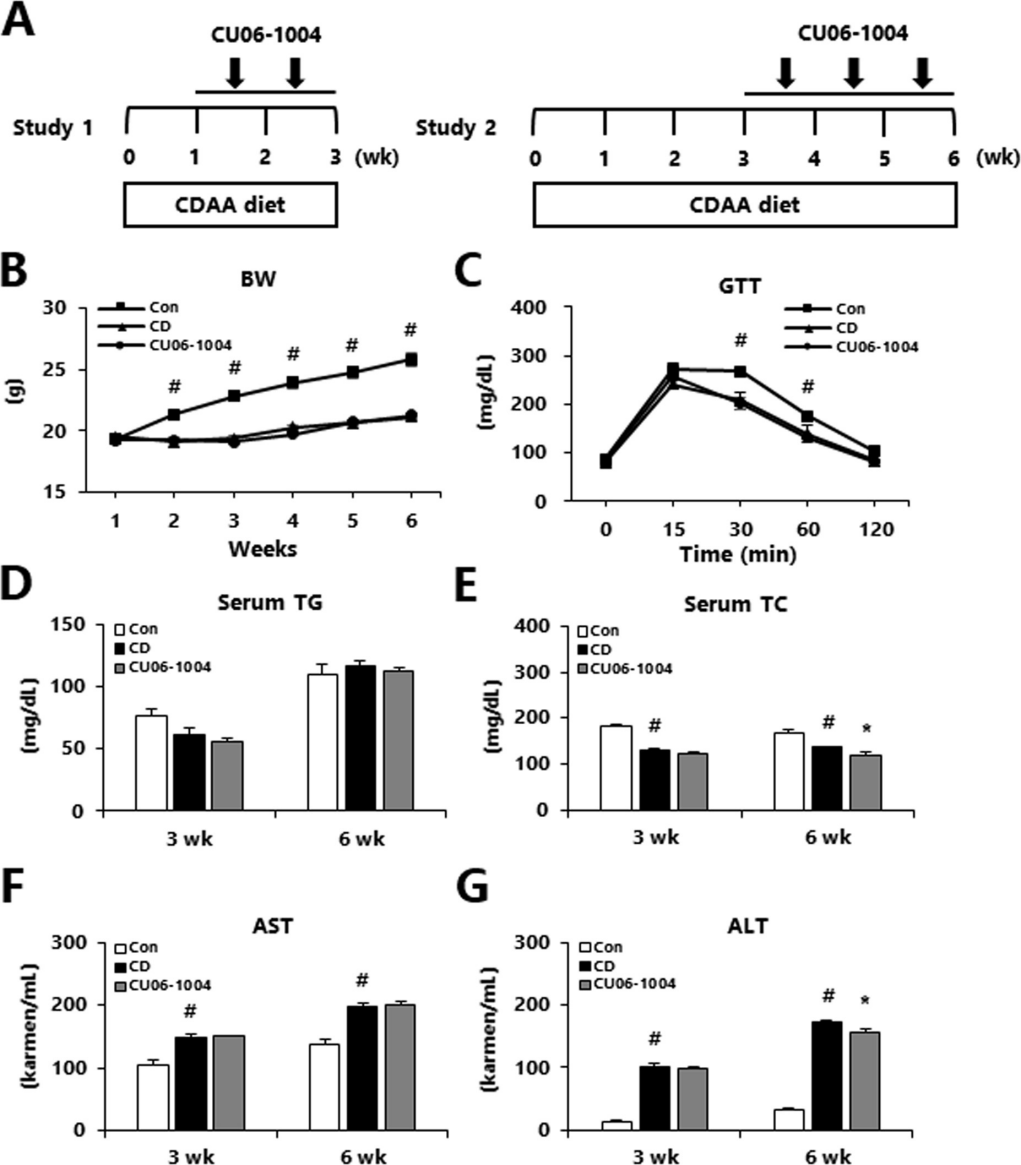
Effects of CU06-1004 on metabolic parameter in mice with choline-deficient L-amino acid (CDAA) diet-induced NASH. (A) Study design to assess the terapeutic effects of CU06-1004. (B) The body weight, (C) Glucose tolerance test (GTT) at 1 week before the end of the experiment, (D) Serum triglycerides (TG), (E) Serum total cholesterol (TC), (F) Aspartate aminotransferase (AST) and (G) Alanine aminotransferase (ALT) levels. Data are presented as the mean ± SEM. ^#^ *p* < 0.05 vs. control group; * *p* < 0.05 vs. CDAA alone group; n = 10 mice/group.

Mild or moderate NASH was induced by feeding mice a CDAA diet for either 3 or 6 weeks. Test mice were treated with oral CU06-1004 for 1 (in study 1) or 3 (in study 2) weeks, starting 2 or 3 weeks before the end of the experiment, respectively (Fig 1A). As expected, the body weight of both groups of mice was increased after a CDAA diet for 3 or 6 weeks relative to control animals, but there was no significant difference in body weight between the mild or moderate NASH groups (S1 Fig, Fig 1B). Additionally, GTT, serum TG, and AST did not differ between mice with mild or moderate NASH (Fig 1C, 1D and 1F; S2 Fig). However, the CU06-1004-treated group had significantly lower serum TC and ALT in the 6-week group, relative to the CD group (Fig 1E and 1G). This indicated that CU06-1004 administration attenuated liver injury in a CDAA-induced model of moderate NASH.

### CU06-1004 prevents hepatic steatosis in a CDAA-induced mouse model of NASH

**Fig 2:**
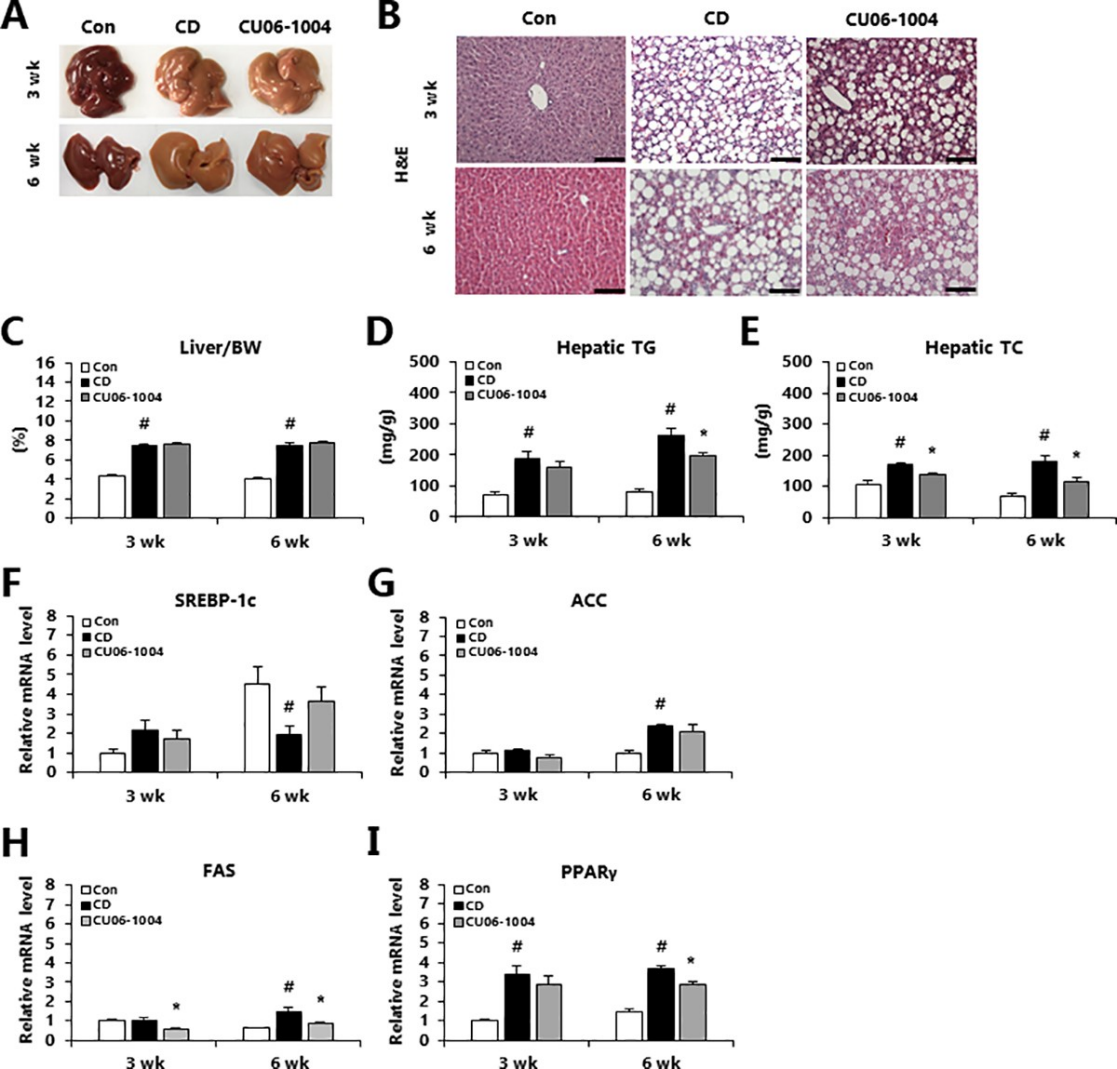
CU06-1004 reduces hepatic lipid accumulation in mice with CDAA diet-induced NASH mice. (A) The representative photographs of livers. (B) Representative H&E-stained liver sections. (C) Ratio of liver weight to body weight, (D) Hepatic triglycerides (TG) and (E) Hepatic total cholesterol (TC). (F-I) Quantitative polymerase chain reaction (qPCR) analysis of mRNA expression in liver of genes involved in lipogenesis. Data are presented as the mean ± SEM. ^#^ *p* < 0.05 vs. control group; * *p* < 0.05 vs. CDAA alone group; n = 10 mice/group (A, C-E); n = 5 mice/group (B); n = 9 mice/group (F-I).

During the early stages of the development of NASH, lipids begin to accumulate in the liver. Therefore, we measured whether treatment with CU06-1004 would impact hepatic lipid accumulation in the CDAA-induced NASH mice. Analysis of liver morphology on HE-stained sections found that the CDAA diet-induced hepatic steatosis. However, the CU06-1004-treated mice had decreased lipid droplets relative to CD mice in both the 3- and 6-week groups (Fig 2A and 2B). The liver-to-body weight ratio was not changed after 3 or 6 weeks of CDAA-induced NASH (Fig 2C). In the 6-week group, CU06-1004 treatment significantly decreased hepatic TG compared to the CD group, however there was no difference in the 3-week group (Fig 2D). The administration of CU06-1004 significantly decreased hepatic TC in mice with both mild and moderate NASH, relative to the CD group (Fig 2E). Therefore, we next investigated lipogenesis-related genes expression. The expression of sterol regulatory element-binding transcription factor-1c (SREBP-1c) and acetyl-CoA carboxylase (ACC) mRNA were not affected by treatment with CU06-1004 (Fig 2F and 2G). However, FAS mRNA expression was significantly decreased in mice with mild or moderate NASH that were treated with CU06-1004 (Fig 2H). The CU06-1004-treated mice in the moderate (6-week), but not mild (3-week), NASH group exhibited significantly decreased expression of peroxisome proliferator-activated receptors γ (PPARγ) relative to the CD group (Fig 2I). These results suggest that CU06-1004 suppressed lipid accumulation via decrease lipogenesis in liver of mice with NASH.

### CU06-1004 attenuates hepatic and systemic inflammation in CDAA-induced NASH

**Fig 3:**
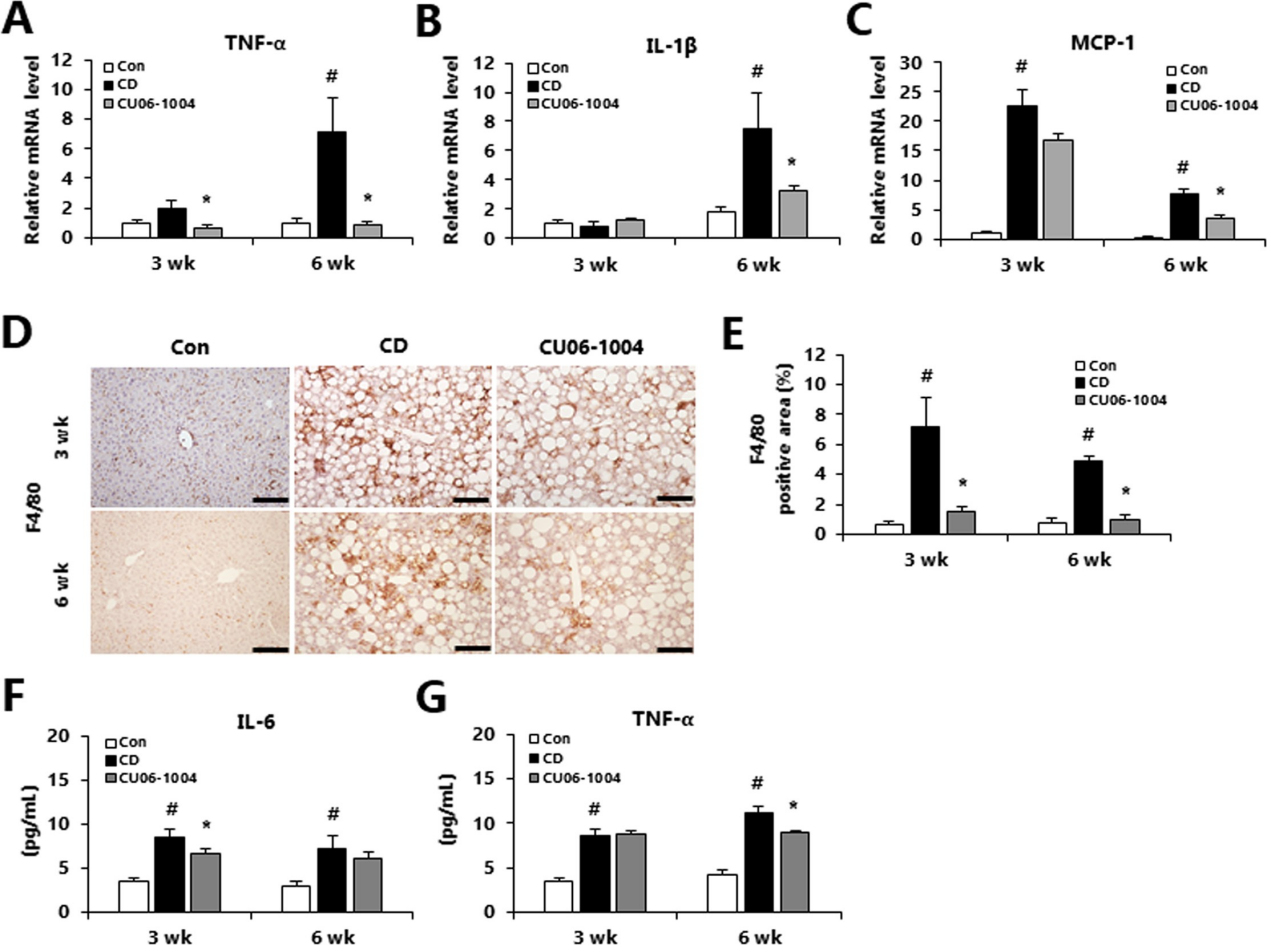
CU06-1004 has anti-inflammatory effects in mice with CDAA diet-induced NASH. (A-C) Quantitative polymerase chain reaction (qPCR) analysis of mRNA expression in liver of genes involved in pro-inflammatory cytokines. (D) Immunohistochemical analysis of F4/80 in liver and (E) quantification of the stain-positive area. (F and G) Serum inflammatory cytokine levels. Data are presented as the mean ± SEM. ^#^ *p* < 0.05 vs. control group; * *p* < 0.05 vs. CDAA alone group; n = 9 mice/group (A-C); n = 8 mice/group (D and E); n = 10 mice/group (F and G).

Hepatic inflammation is an important element of the development of NASH, and LSECs play an important anti-inflammatory role in cooperation with macrophages [23, 24]. In our previous study, we found that treatment with CU06-1004 suppressed the inflammatory response in an animal model of cerebral ischemia [17]. Therefore, we investigated the role of CU06-1004 as a local or systemic modulator of inflammatory changes in NASH mouse model. In the mice with mild (3-week) NASH, CU06-1004 treatment led to significantly decreased mRNA expression of tumor necrosis factor-α (TNF-α) compared with the CD group (Fig 3A). In addition, in mice with moderate NASH (6-week group), treatment with CU06-1004 led to a significant decrease in mRNA expression of M1 macrophage markers such as TNF-α, interleukin-1β (IL-1β), and monocyte chemoattractant protein-1 (MCP-1) compared with the CD group (Fig 3A–3C). Immunohistochemistry staining for F4/80 showed that the CDAA-induced increase in F4/80 positive area was significantly attenuated by treatment with CU06-1004 (Fig 3D and 3E). Moreover, the CU06-1004 group had significantly decreased pro-inflammatory cytokines such as interleukin-6 (IL-6; in mild NASH) and significantly decreased serum TNF-α in moderate NASH (Fig 3F and 3G). Generally, treatment with CU06-1004 appeared to reduce the infiltration of macrophages, thus attenuating systemic inflammation in mice with NASH.

### CU06-1004 suppresses hepatic fibrosis in CDAA-induced NASH mice of mild stage

**Fig 4:**
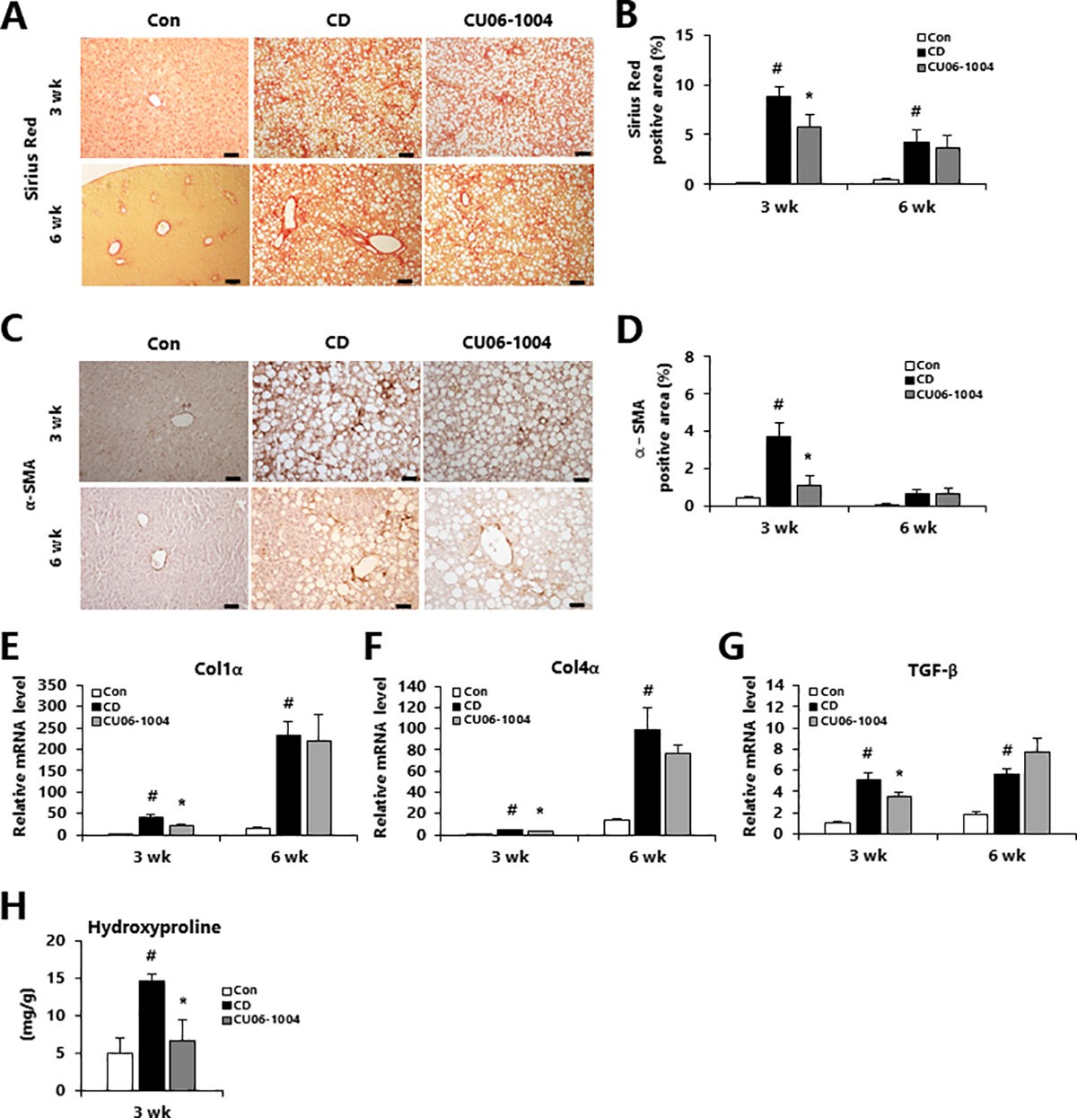
CU06-1004 ameliorates hepatic fibrosis in mice with CDAA diet-induced NASH. (A) Representative images of liver sections stained with picro-sirius red and (B) the stain-positive areas were quantified. (C) Immunohistochemical analysis of hepatic α-SMA content and (D) quantification of the stain-positive area. (E-G) Quantitative polymerase chain reaction (qPCR) analysis of mRNA expression in liver of genes involved in fibrosis-related genes. (H) Hepatic hydroxyproline content. Data are presented as the mean ± SEM. ^#^ *p* < 0.05 vs. control group; * *p* < 0.05 vs. CDAA alone group; n = 4 mice/group (A-D); n = 9 mice/group (E-G); n = 5 mice/group (H).

We next determined whether treatment with CU06-1004 would affect the activation of hepatic stellate cells (HSCs) in the liver of NASH model mice. After 3 weeks of CDAA diet, we found that hepatic the Sirius red staining and α-SMA immunohistochemistry were reduced in CU06-1004-treated mice relative to the CD group (Fig 4A–4D). In addition, the mRNA expression of several fibrosis-related genes such as collagen 1α (Col1α), collagen 4α (Col4α), and transforming growth factor-β (TGF-β) were significantly lower in the CU06-1004 group compared with the CD group (Fig 4E–4G). However, in mice with moderate NASH (6-week), CU06-1004 administration was not associated with a significant change in expression of fibrosis-related genes. Notably, hydroxyproline content was significantly decreased in CU06-1004-treated mice of 3 weeks groups compared with CD mice (Fig 4H). These results suggest that CU06-1004 decreased the accumulation of collagen by inhibiting HSCs in the liver, resulting in decreased deposition of mild fibrillar collagen in the liver.

### CU06-1004 improves LSEC capillarization in mice with mild CDAA-induced NASH

**Fig 5:**
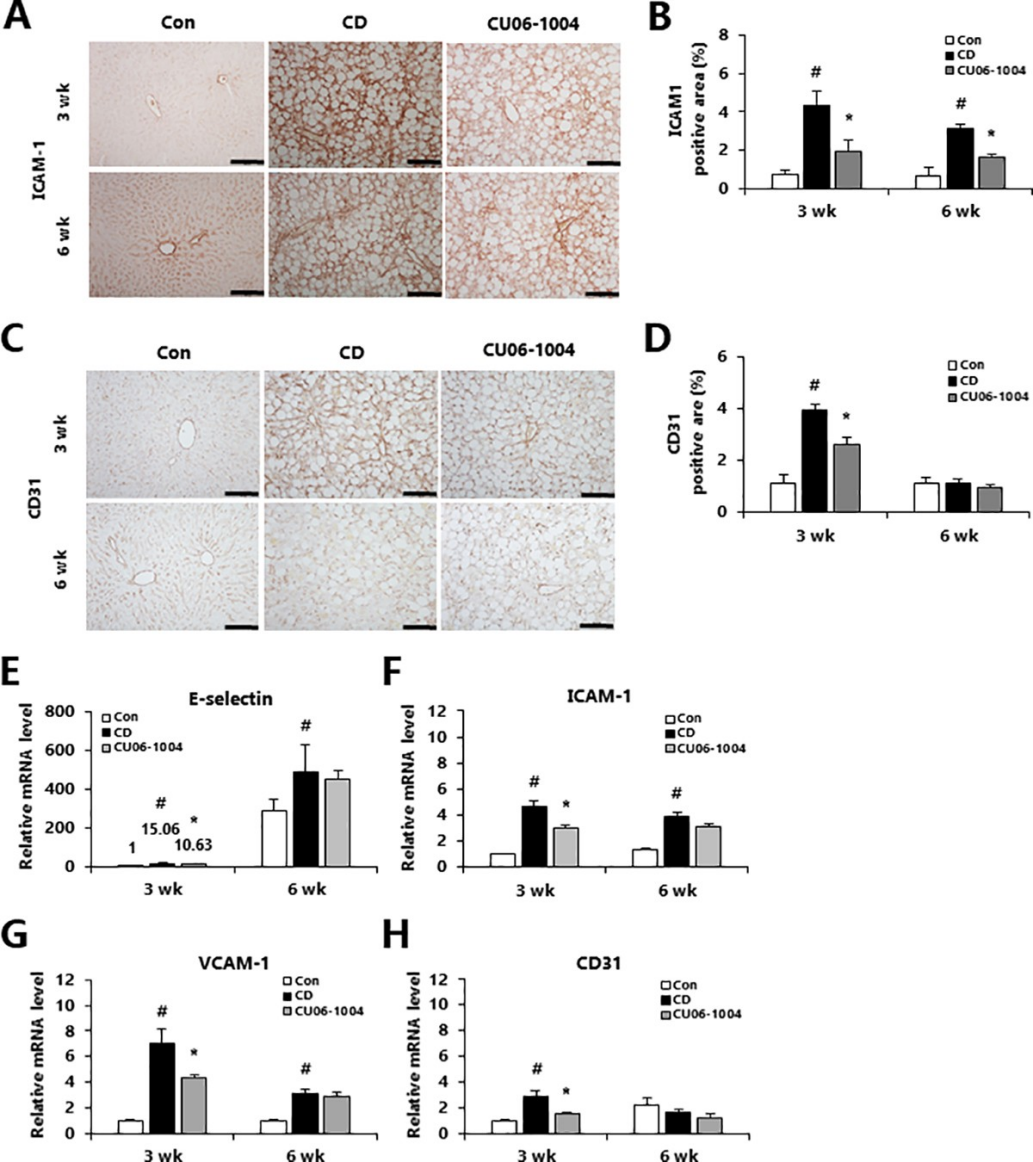
CU06-1004 reduces LSEC capillarization in mice with CDAA diet-induced NASH. (A and C) Immunohistochemical analysis of ICAM-1 and CD31 in liver and (B and D) quantification of the stain-positive area. (E-H) Quantitative polymerase chain reaction (qPCR) analysis of mRNA expression in liver of genes involved in endothelial adhesion molecules-related genes. Data are presented as the mean ± SEM. ^#^ *p* < 0.05 vs. control group; * *p* < 0.05 vs. CDAA alone group; n = 5 mice/group (A-D); n = 9 mice/group (E-H).

LSEC capillarization and dysfunction occur early in the development of metabolic liver diseases [25–29]. In the context of NAFLD, LSECs have higher expression of endothelial adhesion molecules such as E-selectin, intercellular adhesion molecule-1 (ICAM-1), vascular cell adhesion molecule-1 (VCAM-1), and cluster of differentiation 31 (CD31) [30]. Therefore, we investigated changes in expression of ICAM-1 and CD31 through immunohistochemistry staining. The ICAM-1 positive area was significantly decreased in CU06-1004-treated mice compared with CD mice in both mild and moderate NASH (Fig 5A and 5B). However, CD31 expression was only significantly inhibited by CU06-1004 treatment in mice with mild NASH (Fig 5C and 5D). In addition, treatment with CU06-1004 only led to significantly lower mRNA expression of E-selectin, ICAM-1, VCAM-1 and CD31 in mice with mild NASH, relative to the CD groups (Fig 5E–5H). Together, these results suggest that CU06-1004 improved LSEC capillarization most significantly in mice with mild NASH.

## Discussion

In this study, we investigated the therapeutic potential of CU06-1004 in a CDAA-induced mouse model of NASH. We found that CU06-1004 attenuated hepatic steatosis and inflammation in both mild and moderate NASH. Additionally, we observed improvements in the extent of fibrosis and LSEC capillarization in mice with mild NASH that were treated with CU06-1004. In summary, these experiments suggested that CU06-1004 may represent a promising candidate for further investigation as a therapeutic for NASH, with the potential to attenuate hepatic steatosis, inflammation, fibrosis, and changes in LSEC capillarization.

The liver plays a fundamental role in the coordination of metabolism, affecting adipose tissue, skeletal muscle, and performing the essential roles of lipid metabolism. Hepatic steatosis results from imbalanced regulation of lipid accumulation and lipid disposal. Generally, hepatic steatosis occurs at an early stage in the development of NAFLD/NASH [31]. The present study showed that treatment with CU06-1004 reduced the extent of liver injury and the accumulation of lipids in the liver of mice with CDAA-induced NASH, and this protection was correlated with downregulation of genes related to lipogenesis such as FAS and PPARγ. Several studies have shown that expression of FAS mRNA is higher in NAFLD [32, 33]. PPARγ is a transcription factor that regulates lipid metabolism and inflammatory responses in the pathogenesis of NAFLD [34]. In addition, LSECs undergo morphological and functional changes during liver steatosis in the early stages of NAFLD [35]. LSEC capillarization occurs in fatty livers and contributes to increased hepatic vascular resistance [36]. Thus, the regulation of lipogenesis and its transcription factors after treatment with CU06-1004 may be partially due to correction of LSEC dysfunction in mice with CDAA-induced NASH.

Hepatic inflammation is an important factor in the pathogenesis of NASH [37]. Liver injury triggers activation of Kupffer cells, leading to increased expression of pro-inflammatory cytokines and chemokines [38]. LSECs also produce pro-inflammatory mediators in NASH, and the release of inflammatory cytokines from LSECs activates macrophages, further contributing to the inflammatory response [39, 40]. Our immunohistochemistry results show that CU06-1004 treatment was significantly decreased the infiltration of macrophage marker F4/80 in mice with mild or moderate CDAA-induced NASH. Additionally, treatment with CU06-1004 led to decreased expression of pro-inflammatory genes such as TNF-α, IL-1β, and MCP-1 in mice with NASH. TNF-α and IL-6 are key pro-inflammatory factors involved in the development of steatohepatitis [41]. Importantly, in mice with mild or moderate CDAA-induced NASH, treatment with CU06-1004 decreased the concentration of pro-inflammatory cytokines in serum. Previously, we suggested that CU06-1004 decreases inflammation through activation of NF-κB signaling in human brain microvascular endothelial cells (HBMECs) [17]. LSEC activation is characterized by the increased expression of adhesion molecules such as E-selectin, ICAM-1, and VCAM-1, which influence cell-to-cell interactions and are regulated by inflammatory cytokines [42, 43]. We found that treatment with CU06-1004 downregulated the expression of E-selectin, ICAM-1, and VCAM-1. This finding is consistent with our previous study, which found that CU06-1004 attenuates the expression of adhesion molecules after ischemic reperfusion injury [17]. These results suggest that CU06-1004 may have value in attenuating hepatic and systemic inflammation and in decreasing the expression of cell adhesion molecules by LSECs in mice with CDAA-induced NASH.

Liver fibrosis is a major pathological process in NASH that is mediated by HSC activation. The activation of HSCs induces changes in morphology and extracellular matrix, as indicated by increases in the Sirius red and α-SMA staining [44]. CU06-1004 suppresses fibrosis in mice with mild CDAA-induced NASH mice, as evidenced by decreased staining with Sirius red and α-SMA as well as decreased expression of pro-fibrotic genes such as Col1α, Col4α, and TGF-β. These results indicate that CU06-1004 plays anti-fibrogenic effects by causing decreased activation of HSC. Furthermore, in healthy livers, LSECs prevent activation of HSCs and thus have anti-fibrogenic properties [45]. However, capillarized LSECs release inflammatory mediators and contribute to the recruitment of macrophages and HSCs, thus promoting inflammation and fibrosis [46]. CD31 is a common marker for LSEC capillarization that has been used to assess for increased LSEC capillarization in NASH [5]. In mice with mild CDAA-induced NASH, treatment with CU06-1004 decreased the expression of CD31, suggesting that it attenuated sinusoidal capillarization. Therefore, we speculate that the anti-fibrotic effects of CU06-1004 are at least partially achieved through preventing capillarization of LSECs.

Altogether, our results demonstrated that CU06-1004 could be a valuable therapeutic candidate with the potential to attenuate hepatic steatosis, inflammation, fibrosis, and LSEC capillarization in mice with CDAA-induced NASH. This hepatoprotective effect of CU06-1004 was correlated to the inhibition of liver TG content, pro-inflammatory cytokines and hepatic collagen deposition. Therefore, future studies will need to examine the effects and molecular mechanisms of CU06-1004 in parenchymal and non-parenchymal cells in mouse models of NASH.

## Supporting information

S1 Fig Body weight in mice with CDAA diet-induced NASH for 3 weeks. Data are presented as the mean ± SEM. ^#^ *p* < 0.05 vs. control group; n = 10 mice/group.
(DOCX)

S2 Fig Glucose Tolerance Test (GTT) in mice CDAA diet-induced NASH at 3 weeks. Data are presented as the mean ± SEM. ^#^ *p* < 0.05 vs. control group; n = 5 mice/group.
(DOCX)
