# Correction: The endothelial dysfunction blocker CU06-1004 ameliorates choline-deficient L-amino acid diet-induced non-alcoholic steatohepatitis in mice

**DOI:** 10.1371/journal.pone.0249747

**Published:** 2021-04-05

**Authors:** Cho-Rong Bae, Haiying Zhang, Young-Guen Kwon

There are errors in the Funding statement. The correct Funding statement is as follows: CURACLE Co, Ltd., provided support for the study in the form of salary for HZ, and provided the CU06-1004 sample. The specific roles of this author are articulated in the ‘author contributions’ section. This research was supported by the National Research Foundation of Korea (NRF) grant funded by the Korea government (MSIT, 2019R1C1C1003754), and the Ministry of Science, ICT & Future Planning (MSIP; NRF-2019R1A2C3007142). This work was also supported in part by the Brain Korea 21 (BK21) PLUS program. The funders had no role in study design, data collection and analysis, decision to publish, or preparation of the manuscript.
