# Supplementary material for: The endothelial dysfunction blocker CU06-1004 ameliorates choline-deficient L-amino acid diet-induced non-alcoholic steatohepatitis in mice
Source: PLoS One. 2020 Dec 4;15(12):e0243497. doi: 10.1371/journal.pone.0243497 (PMC7717513; doi:10.1371/journal.pone.0243497)
Supplement: S1 Fig — Data are presented as the mean ± SEM. # p < 0.05 vs. control group; n = 10 mice/group. (DOCX) [file pone.0243497.s001.docx]

**
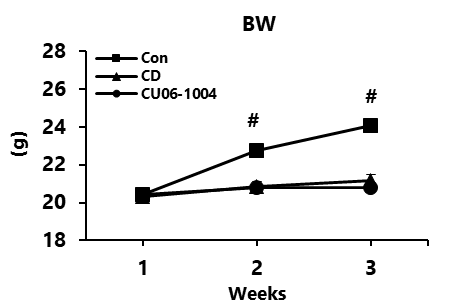
**

**S1 Fig. Body weight in mice with CDAA diet-induced NASH for 3 weeks.**

Data are presented as the mean ±SEM. ^#^ *p* < 0.05 vs. control group; n = 10 mice/group.
